# Conservative management of avascular necrosis of the metacarpal head: A case report and brief review: Retraction

**DOI:** 10.1097/MD.0000000000026376

**Published:** 2021-06-18

The article, “Conservative management of avascular necrosis of the metacarpal head: A case report and brief review”,^[1]^ which published in Volume 100, Issue 20 of *Medicine*, is being retracted. The authors did not receive approval for publishing a translated version of the article, which was previously published^[2]^ in Chinese in *Chinese Journal of Orthopaedics* and *Medicine* was not informed that the submission was a translated version of a previously published article.
